# Septic arthritis caused by *Candida dubliniensis* following arthroscopic surgery

**DOI:** 10.1016/j.mmcr.2024.100634

**Published:** 2024-02-16

**Authors:** Nayla Azanki Hatem, Alessandro C. Pasqualotto, Cecília Bittencourt Severo, Rafael Hannaui Bastos, Rafael de Luca de Lucena, Cezar Vinícius Würdig Riche

**Affiliations:** a Santa Casa de Misericordia de Porto Alegre, Porto Alegre, Brazil; b Federal University of Health Sciences of Porto Alegre (UFCSPA), Porto Alegre, Brazil; c Lutheran University of Brazil (ULBRA), Canoas, Brazil

**Keywords:** Septic arthritis, *Candida dubliniensis*, Fungal arthritis, *Candida albicans*

## Abstract

A 37-year-old immunocompetent man was admitted to the emergency department due to recurrent pain and oedema of his right knee. Two months earlier, he had undergone surgery to repair his meniscus. Arthroscopic joint lavage was performed and *Candida dubliniensis* was recovered in culture. The authors describe the first case of septic arthritis caused by *Candida dubliniensis*.

## 1 Introduction

Fungal septic arthritis is a rare but severe infection, and it is most caused by *Candida* species, especially in immunocompromised patients [1]. Little is known about these infections and most reports are limited to individual case descriptions and relatively small case series [2]. Additionally, the underlying mechanisms responsible for *Candida* arthritis are not fully comprehended, and initial reports indicates that it frequently arises as a complication of disseminated candidosis, being *C. albicans* is the most isolated species [3].

Incidence of fungal arthritis is relatively low due to *Candida* species other than *C. albicans*, accounting for less than 1% of all cases [4]. *Candida dubliniensis* represents only a small percentage of *Candida* isolates recovered from clinical samples, and despite the effort that has been expended to identify this species, a definitive assessment of the prevalence of this type is still lacking. Here we describe the first reported case of septic arthritis by *C. dubliniensis*.

## 2 Case presentation

A 37-year-old man with no relevant comorbidities was admitted to the hospital due to recurrent pain and oedema of his right knee. His past medical history was marked by a meniscus surgery repair performed in January 2023, as a consequence of a tear incurred during a Jiu-Jitsu training. He denied alcohol or intravenous drug abuse.

Approximately 50 days post-surgery, the patient presented to the outpatient orthopaedic clinic reporting painful swelling and oedema in his right knee, accompanied by the drainage of fluid from the joint. He did not present with a fever or any other systemic signs or symptoms. He was then referred to the emergency department by the orthopaedist (Day 0). Blood tests at admission revealed a C-reactive protein of 15mg/L (reference range <5mg/L), erythrocyte sedimentation rate of 2mm (reference range <15mm), and white blood cell (WBC) of 11,980/μL (reference range 3600-11,000/μL), with 72% of neutrophils. Vital signs were normal. Oxacillin and gentamicin were started empirically.

The patient underwent arthroscopic joint lavage on the same day of admission. No cell count was obtained. The Gram stain and acid-fast stain of the fluid did not reveal any presence of bacteria or mycobacteria. On the following day (Day 1), yeast growth was observed in a culture obtained from the knee effusion. Consequently, intravenous fluconazole was initiated at a daily dose of 400mg.

On day 3, MALDI-TOF analysis successfully identified *Candida dubliniensis* in the synovial fluid (log score value of >2.0), leading to the discontinuation of antibiotics. Further laboratory testing confirmed negative serologies for hepatitis A, B, and C, as well as for HIV infection. Blood cultures were negative. With the diagnosis of septic arthritis caused by *C. dubliniensis* established, the patient underwent a full 8-day course of intravenous antifungal treatment. Subsequently, he was discharged (Day 8), and prescribed oral fluconazole at a daily dosage of 300mg. Antifungal susceptibility testing was not performed.

On day 20, the patient returned to the emergency department due to worsening oedema, hyperaemia, and persistent knee pain. A new arthroscopic lavage was performed, and cultures were obtained. Anidulafungin 100mg once daily, was initiated. Upon direct microscopy of the synovial fluid from the right knee, yeast colonies were detected, and further analysis via MALDI-TOF confirmed the presence of *C. dubliniensis* once again. Susceptibility antifungal test was performed according to current protocols of the Brazilian Committee on Antimicrobial Susceptibility Testing (BrCAST/EUCAST) and presented susceptibility to fluconazole (MIC of 0.25μg/ml) and micafungin (MIC of 0.015μg/ml).

The patient received anidulafungin therapy for four weeks and two more arthroscopic joint lavage were performed, leading to significant improvements in pain and oedema. No further swelling or fluid drainage was observed. Subsequently, the patient was discharged (Day 48) with an oral fluconazole regimen of 300mg daily for a duration of two weeks, and there were no subsequent relapses observed.

## 3 Discussion

*Candida* species are a leading cause of fungal infections in humans, especially in those with impaired immunity, often referred to as opportunistic pathogens [2]. These yeasts can result in a wide range of diseases, from mild, superficial conditions like vaginal and oral thrush to severe systemic infections like candidaemia. Although the *Candida* genus comprises more than 200 species, only a limited number of them have been associated with human infections and *C. albicans* contribute to the highest incidence of *Candida* septic arthritis cases [5].

Fungal arthritis is uncommon in immunocompetent patients and overall incidence is relatively low due to non-*C. albicans Candida* species, which is less than 1% of all cases [4]. Most patients with fungal infections are immunosuppressed with predisposing factors (e.g., autoimmune disease, HIV infection, chronic steroid use, cancer cachexia, or recreational drugs) [4,6]. However, healthy hosts can also develop fungal musculoskeletal infections due to nosocomial exposures, such as prior surgery of placement of indwelling foreign bodies [7].

The apparent pathogenesis of most cases of *Candida* arthritis is that of haematogenous dissemination to the joint [2]. Our patient was immunocompetent, and the risk factor identified was prior knee surgery. Whilst haematogenous dissemination is considered the predominant infection mechanism, direct inoculation during surgery or intra-articular infection is also a plausible route for joint infection [2,3].

This is the first known reported case of septic arthritis caused by *Candida dubliniensis,* observed in both immunocompromised and immunocompetent individuals. This *Candida* species was first identified in 1995 and the first isolates were recovered from oral samples of HIV-positive and AIDS patients with recurrent oral candidosis [8,9]. Despite this discovery, research on the risk factors and prevalence of this species' diseases in humans remains limited. The majority of *C. dubliniensis* isolates have been found in the respiratory tract, most often recovered from the oral cavity of HIV-infected patients (1–48%) [10]. It also has been recovered from HIV-negative and healthy persons (2–9%) and isolated from other body sites (respiratory tract, skin, urinary tract, or central nervous system) [10,11]. Recent findings suggest its potential involvement in invasive infections, and *C. dubliniensis* has been described specifically as a cause of central venous catheter-associated infections, endocarditis, and endophthalmitis [12].

*C. dubliniensis* is closely related to *C. albicans*, and both species share many phenotypic characteristics including the ability to produce hyphae and chlamydospores [13]. However, *C. dubliniensis* is responsible for fewer infections in humans, and is rare for patients colonized with this *Candida* species to develop candidaemia [11]. Studies have focused on understanding the limited ability of this organism to cause invasive disease. It has demonstrated that the genome of *C. dubliniensis* lacks crucial virulence genes related to hyphae and exhibits restricted capacity for yeast-to-hyphal transformation. This may impact its potential to invade deeper tissue [11,13].

Until recently, identification of *C. dubliniensis* was difficult in the clinical laboratories and due to phenotypic similarity between *C. dubliniensis and C. albicans, C. dubliniensis* was previously misidentified as *C. albicans* [5]. However, due to an improvement in identifications methods via species-specific polymerase chain reaction and MALDI-TOF MS usage, there has been an increase in reported cases caused by this species [13,14]. In our case, MALDI-TOF was essential to establish the diagnosis and provide a more definitive and rapid identification.

Management of *Candida* septic arthritis often requires antifungal therapy and surgical approach [15]. Because the clinical manifestations and inflammatory markers are not specific for *Candida* arthritis, arthrocentesis or arthroscopy is warranted for the definitive diagnosis [2]. The vast majority of *C. dubliniensis* isolates identified are known to be susceptible to a wide range of antifungal agents [16], however, reduced susceptibility to azole antifungal drugs has been observed in clinical isolates and can be readily induced in vitro [14,16]. In this case, considering the isolate's in vitro susceptibility to fluconazole, the relapse seen on day 20 was attributed to the necessity of a new arthroscopic lavage, rather than a failure of fluconazole treatment. Both surgical intervention and pharmacological management were crucial for achieving successful treatment.

## 4 Conclusion

This is the first report in the literature of *C. dubliniensis* causing septic arthritis, an infrequent fungal pathogen, especially in an immunocompetent individual. This unusual *Candida* species may be a potential causative agent for osteoarticular infections, particularly in patients with a history of previous joint surgery. A high index of suspicion is essential to establish the correct diagnosis and the timely is of great importance to help initiate antifungal therapy promptly. The successful treatment could prevent irreversible joint destruction, as well as preserve articular function.

## Conflict of interest

Prof. Pasqualotto has received research grants from 10.13039/100005564Gilead Sciences, 10.13039/100004319Pfizer, 10.13039/100030732MSD, IMMY, and 10.13039/100011893PAHO. He has consulted and/or given paid talks on behalf of Gilead Sciences, Pfizer, Knight, MSD, Teva, IMMY, Astellas, and Sandoz. All other authors have no conflict of interest to declare.

## Ethical form

The authors have obtained written and signed consent to publish the case report.

## CRediT author statement

Nayla Azanki Hatem: Conceptualization, Methodology, Visualization, Writing – original draft. Alessandro C. Pasqualotto: Writing – review & editing. Cecília Bittencourt Severo: Writing – review & editing. Rafael Hannaui Bastos: Writing – review & editing. Rafael de Luca de Lucena: Writing – review & editing. Cezar Vinícius Würdig Riche: Conceptualization, Methodology, Visualization, Supervision, Writing – review & editing.
